# Vitamin D Supplementation in Children with Asthma: An Umbrella Review

**DOI:** 10.3390/nu18101560

**Published:** 2026-05-14

**Authors:** Jianzhao Liu, Yujun Long, Zhirong Yang

**Affiliations:** 1 Department of Biomedical Engineering, Southern University of Science and Technology, Shenzhen 518055, China; 2 Department of Computational Biology and Medical Big Data, Faculty of Computer Science and Artificial Intelligence, Shenzhen University of Advanced Technology, Shenzhen 518107, China; 3 Shenzhen Institutes of Advanced Technology, Chinese Academy of Sciences, Shenzhen 518055, China; 4 University of Chinese Academy of Sciences, Beijing 100049, China; 5 Center for AI in Medicine, Artificial Intelligence Research Institute, Shenzhen University of Advanced Technology, Shenzhen 518107, China

**Keywords:** vitamin D, children, asthma, umbrella review

## Abstract

Background: Growing evidence suggests that vitamin D plays a role in the pathophysiology of childhood asthma. However, its effectiveness in reducing asthma exacerbations and improving asthma-related outcomes remains controversial. Methods: We systematically searched PubMed, Embase, and the Cochrane Library from inception to 25 February 2026. Meta-analyses of randomized controlled trials (RCTs) evaluating the effects of vitamin D supplementation on any health outcomes in children with asthma were included. Methodological quality was assessed using the AMSTAR 2 tool. The credibility of evidence was evaluated using pre-specified evidence classification criteria, and the certainty of evidence was graded using the GRADE approach. Results: A total of 14 systematic reviews were included, of which one was rated as high quality, six as low quality, and seven as critically low quality according to AMSTAR 2. Vitamin D supplementation significantly increased serum 25-hydroxyvitamin D levels in children with asthma (MD = 10.68 ng/mL; 95% CI, 6.30 to 15.05; *n* = 8 RCTs), although the evidence was of low credibility (class IV) and very low certainty. No significant improvements were observed in Childhood Asthma Control Test scores (MD = 0.15; 95% CI, −0.43 to 0.74; *n* = 3 RCTs; class V; moderate), overall asthma exacerbations (RR = 0.84; 95% CI, 0.65 to 1.08; *n* = 11 RCTs; class V; low), or lung function as measured by percent predicted forced expiratory volume in 1 second (SMD = 0.49; 95% CI, −0.05 to 1.04; *n* = 5 RCTs; class V; moderate). One meta-analysis suggested a possible reduction in asthma recurrence (RR = 0.53; 95% CI, 0.35 to 0.79; *n* = 6 RCTs; class IV; moderate). Conclusions: This umbrella review found no convincing evidence that vitamin D supplementation improves asthma control, reduces exacerbations, or enhances lung function in children with asthma, despite its effect on increasing serum 25-hydroxyvitamin D levels and a possible benefit for asthma recurrence. However, these findings should be interpreted with caution, considering that the available evidence was limited by generally low methodological quality, substantial overlap among meta-analyses, and incomplete reporting of clinically relevant modifiers.

## 1. Introduction

Asthma is one of the most common chronic respiratory diseases affecting individuals across all age groups, with a peak incidence occurring in childhood [1]. It is characterized by chronic airway inflammation, variable airflow limitation, and recurrent symptoms such as wheeze, cough, and shortness of breath [2]. Despite advances in pharmacological management, a substantial proportion of children continue to experience poor symptom control and recurrent exacerbations, highlighting the need for safe and effective adjunctive strategies to improve asthma outcomes.

Vitamin D has attracted increasing attention as a potentially modifiable factor in asthma because of its immunomodulatory and anti-inflammatory properties. Beyond its established role in calcium and phosphate homeostasis, vitamin D may influence several pathways relevant to asthma pathogenesis. Experimental and mechanistic studies suggest that vitamin D can suppress type 2 helper (Th2) cell responses, which play a central role in the pathophysiology of asthma [3]. In addition, vitamin D inhibits B-cell proliferation and differentiation into plasma cells, leading to reduced secretion of immunoglobulin E (IgE), a key mediator of allergic airway inflammation [4]. Vitamin D may also affect airway remodeling, susceptibility to respiratory infections, and responsiveness to corticosteroid therapy, all of which are clinically relevant in pediatric asthma. Observational studies have reported that lower serum 25-hydroxyvitamin D (25(OH)D) concentrations are associated with a higher risk of asthma, poorer asthma control, and increased exacerbation risk in children [5,6].

However, evidence regarding the clinical benefits of vitamin D supplementation for childhood asthma remains inconsistent. Earlier studies and meta-analyses suggested a favorable effect of vitamin D supplementation [7,8], whereas several subsequent randomized controlled trials (RCTs) failed to demonstrate significant benefits in children with asthma [9,10]. Several factors may contribute to these inconsistencies, including differences in baseline vitamin D status, asthma severity, supplementation dose and regimen, treatment duration, and outcome definitions. Given these conflicting findings, a comprehensive and up-to-date synthesis of the available evidence, together with a rigorous assessment of evidence credibility and certainty, is warranted.

Existing reviews have primarily focused on pooled efficacy estimates, but have paid less attention to differences in methodological quality across reviews, the degree of overlap among included primary trials, and the overall credibility and certainty of the reported associations. As a result, it remains unclear whether the apparent benefits of vitamin D supplementation are consistent, robust, and clinically actionable. An umbrella review can address these limitations by synthesizing evidence across published reviews, systematically assessing methodological quality, evaluating the certainty of evidence, classifying the credibility of associations, and quantifying overlap across reviews. Together, these approaches provide insight into the reliability, independence, and potential redundancy of the existing evidence base and may generate more actionable conclusions for clinical practice and future research.

Therefore, we conducted this umbrella review to evaluate the efficacy of vitamin D supplementation as an adjunctive therapy in children with asthma. To our knowledge, this is the first comprehensive synthesis of existing evidence on this topic and aims to provide a robust evidence base to inform clinical practice and guideline development.

## 2. Methods

### 2.1. Study Design

This umbrella review was reported following the Preferred Reporting Items for Overviews of Reviews (PRIOR) statement for healthcare interventions [11]. The protocol was prospectively registered in the International Prospective Register of Systematic Reviews (PROSPERO; Registration ID: CRD420261294673).

### 2.2. Literature Search

A comprehensive systematic literature search was performed in PubMed, Embase, and the Cochrane Library, using predefined search terms, from database inception to 25 February 2026. Full search strategies are available in Supplementary Table S1.

### 2.3. Inclusion and Exclusion Criteria

The systematic reviews incorporating meta-analyses were included if they satisfied the following criteria: (1) focusing on children (0–18 years) with asthma; (2) evaluating vitamin D supplementation versus placebo, regardless of the type, dosage, or duration; (3) reporting health-related outcomes such as asthma control, pulmonary function tests, serum vitamin D levels, or inflammatory biomarkers; and (4) including randomized controlled trials.

Reviews that exclusively focused on the prevention of childhood asthma were excluded. We did not exclude studies based on the publication language.

All eligible meta-analyses within the included reviews were initially considered. To avoid duplication of evidence, when multiple meta-analyses addressed the same health outcome, only one was selected for GRADE assessment. In such cases, the most recent and/or largest meta-analysis was selected (Supplementary Methods S1), on the assumption that newer and larger meta-analyses were more likely to encompass the same randomized controlled trials as earlier and smaller ones addressing the same research question [12,13]. AMSTAR 2 was used to evaluate the methodological quality of all included systematic reviews; however, it was not applied as a formal prespecified criterion for selecting the meta-analysis for GRADE assessment. This approach was adopted to prioritize the most up-to-date and comprehensive direct evidence synthesis while minimizing redundant grading of overlapping review-level evidence. We excluded network meta-analyses because we prioritized evidence derived from direct comparisons.

The literature screening was performed independently by two authors (J.L. and Y.L.), with any disagreements resolved through consensus with a third reviewer (Z.Y.).

### 2.4. Data Extraction

A standardized extraction template was used to summarize review characteristics. For each included study, data on the first author, year of publication, intervention and comparator, outcomes assessed, study duration, sample size, age range, and the number and design of RCTs were collected. Summary effect estimates, including mean difference (MD), standardized mean difference (SMD), odds ratio (OR), or risk ratio (RR), together with their corresponding 95% confidence intervals (CIs) and I^2^ statistics, were extracted.

Data extraction was performed by one reviewer (J.L.) and independently verified by a second reviewer (Y.L.). Any disagreements were resolved through discussion, with a third reviewer (Z.Y.) consulted when necessary.

### 2.5. Overlap of Primary Trials Across Included Reviews

To assess the degree of overlap among the included systematic reviews, we applied the Corrected Covered Area (CCA) method [14]. A citation matrix was constructed with unique randomized controlled trials listed in rows and included reviews listed in columns. The CCA was calculated using the following formula:

$$\mathrm{CCA}=\frac{N-r}{r\times c-r}$$

where *N* is the total number of trials included across all reviews (counting repeated occurrences), *r* is the number of unique primary studies, and *c* is the number of included reviews. According to established criteria, overlap was categorized as slight (0–5%), moderate (6–10%), high (11–15%), or very high (>15%).

### 2.6. Quality, Certainty and Credibility Assessment

The methodological quality of the included reviews was independently evaluated by two reviewers (J.L. and Y.L.) using the validated A Measurement Tool to Assess Systematic Reviews 2 (AMSTAR 2) [15]. The tool assesses 16 methodological domains, including study design, literature search, statistical methods applied in meta-analyses, interpretation of findings, consideration of risk of bias, exploration and explanation of heterogeneity, and assessment of publication bias. Based on the presence of critical and non-critical weaknesses, reviews with critical flaws were judged to be of low or critically low quality, whereas those with no or only one non-critical weakness were classified as high quality.

The certainty of evidence for each distinct pooled estimate was evaluated using the Grading of Recommendations, Assessment, Development and Evaluation (GRADE) approach and categorized into four levels: high, moderate, low, or very low [16,17]. Within this framework, evidence derived from randomized controlled trials is initially rated as high certainty and may be downgraded according to five prespecified domains, including risk of bias, inconsistency, indirectness, imprecision, and publication bias [18].

In addition, the credibility of evidence for each outcome was classified into five categories according to evidence classification criteria used in previous umbrella reviews: convincing (class I), highly suggestive (class II), suggestive (class III), weak (class IV), or no evidence (class V) [12,19]. These classifications were determined based on predefined criteria, as detailed in Supplementary Table S2.

### 2.7. Data Analysis

Pooled effect estimates and heterogeneity were recalculated using a random-effects model, if I^2^ statistics were not reported in the original meta-analyses. Data on publication bias were retrieved from the included studies; when unavailable, publication bias was evaluated using funnel plot inspection and Egger’s regression test. A *p*-value < 0.05 was considered statistically significant.

## 3. Results

### 3.1. Literature Retrieval

**Figure 1:**
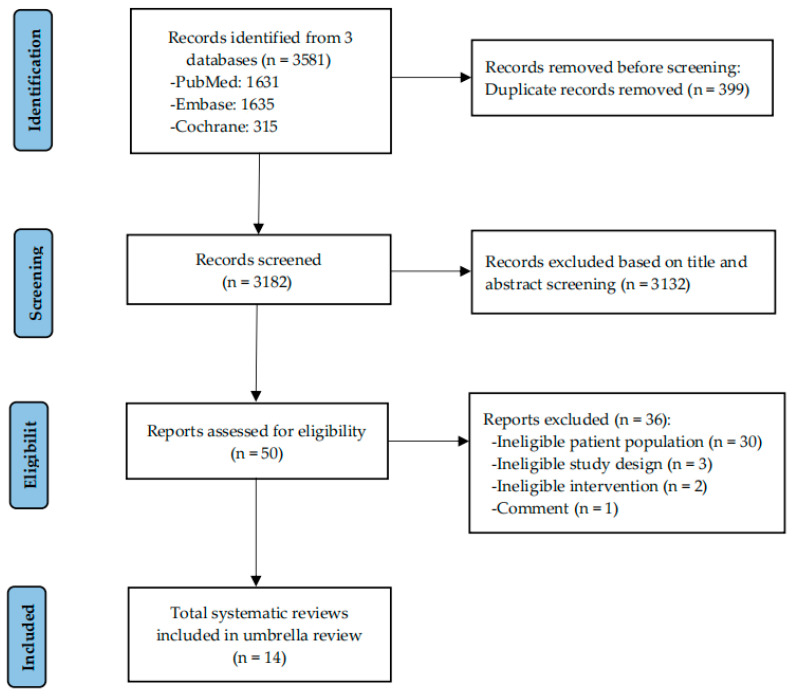
Flowchart of the study selection for the umbrella review.

A total of 3581 records were identified through database searches. After duplicate removal and title/abstract screening, 50 full-text reports were assessed for eligibility, and 14 systematic reviews were ultimately included in this umbrella review [20,21,22,23,24,25,26,27,28,29,30,31,32,33]. The study selection process is illustrated in Figure 1 using a PRIOR flow chart.

The eligible articles were published between 2014 and 2024, with 10 studies published within the past five years. The number of RCTs included in each review ranged from 3 to 18, with sample sizes ranging from 149 to 1579 participants. Regarding methodological assessment of the primary trials, 12 studies applied the Cochrane risk of bias assessment tool. Detailed characteristics of the reviews included are summarized in Table 1.

### 3.2. Quality Assessment

The results of the methodological quality assessments were displayed in Table 2. Only one review was rated as high methodological quality [21], while all remaining reviews were assessed as having low to critically low quality. Regarding individual AMSTAR 2 criteria, key concerns emerged primarily in items 2, 7 and 13. Specifically, 36% of the reviews did not report protocol registration (item 2), 86% failed to provide a list of excluded studies with justification (item 7), and 29% did not adequately account for the risk of bias of individual studies when interpreting or discussing the review findings (item 13).

One health outcome was supported by suggestive evidence (class III), whereas all other outcomes were classified as weak (class IV) or non-significant evidence (class V) (Supplementary Table S3).

### 3.3. Overlap of Included Studies

**Figure 2:**
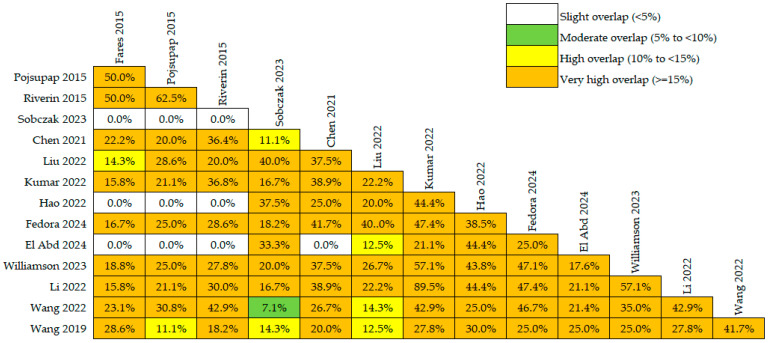
Heatmap of pairwise overlap of primary studies across the included systematic reviews [21,22,23,24,25,26,27,28,29,30,31,32,33,34].

Among the 14 included systematic reviews, 26 unique primary randomized controlled trials were identified (Supplementary Table S4) [7,8,9,10,34,35,36,37,38,39,40,41,42,43,44,45,46,47,48,49,50,51,52,53,54,55]. The overlap analysis showed a substantial degree of overlap across reviews, indicating that many reviews relied on the same set of primary trials. The calculated CCA was 28.4%, suggesting very high overlap among the included reviews. A graphical overview of pairwise overlap across reviews is shown in Figure 2.

### 3.4. Asthma Control and Exacerbation Related Outcomes

Vitamin D supplementation showed no statistically significant effect on childhood asthma control (RR = 1.01; 95% CI, 0.91 to 1.13; *n* = 4 RCTs; credibility of evidence: class V; certainty of evidence: high). Likewise, no significant improvement was observed in the Childhood Asthma Control Test (C-ACT) score (MD = 0.15; 95% CI, −0.43 to 0.74; *n* = 3 RCTs; class V; moderate).

No significant association was observed for asthma exacerbations overall (RR = 0.84; 95% CI, 0.65 to 1.08; *n* = 11 RCTs; class V; low). Vitamin D supplementation was also not significantly associated with asthma exacerbations requiring systemic corticosteroids (OR = 1.28; 95% CI, 0.83 to 1.97; *n* = 9 RCTs; class V; moderate) or exacerbations requiring emergency department visits or hospitalization (RR = 1.01; 95% CI, 0.91 to 1.12; *n* = 5 RCTs; class V; moderate). However, one meta-analysis suggested that vitamin D supplementation was associated with a reduced asthma recurrence rate (RR = 0.53; 95% CI, 0.35 to 0.79; *n* = 6 RCTs; class IV; moderate).

### 3.5. Lung Function

Low-certainty evidence suggested that vitamin D supplementation had no significant effect on percent predicted forced expiratory volume in 1 second (FEV1%) (SMD = 0.49; 95% CI, −0.05 to 1.04; *n* = 5 RCTs; class V) or fractional exhaled nitric oxide (FeNO) levels (MD = −3.95; 95% CI, −22.87 to 14.97; *n* = 2 RCTs; class V; very low) in children with asthma. No significant association was also observed for the FEV1/FVC ratio (MD = −0.86; 95% CI, −3.52 to 1.79; *n* = 2 RCTs; class V; moderate). In contrast, moderate certainty evidence indicated that vitamin D supplementation was associated with a change in percent predicted forced vital capacity (FVC%) (MD = −5.01; 95% CI, −9.99 to −0.02; *n* = 2 RCTs; class IV).

### 3.6. Other Outcomes

**Figure 3:**
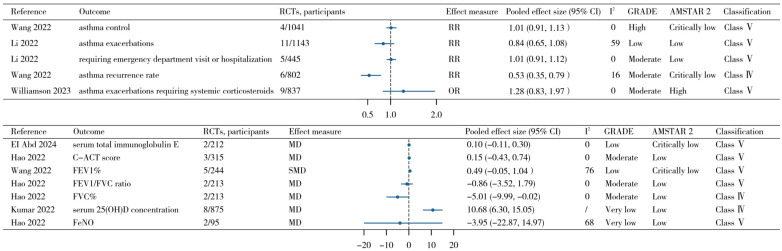
Effects of vitamin D supplementation in children with asthma [21,22,23,29,30,32]. Abbreviations: C-ACT score: Childhood Asthma Control Test score; FEV1%: forced expiratory volume in 1 s percent predicted; FVC%: forced vital capacity percent; FeNO: fractional exhaled nitric oxide.

Vitamin D supplementation significantly increased serum 25(OH)D levels in children with asthma (MD = 10.68 ng/mL; 95% CI, 6.30 to 15.05; *n* = 8 RCTs; class IV; very low). No significant effect was observed on serum total immunoglobulin E (IgE) levels (MD = 0.10; 95% CI, −0.11 to 0.30; *n* = 2 RCTs; class V; low). All pooled meta-analytic estimates extracted from the included reviews are summarized in Figure 3 and Supplementary Table S5.

## 4. Discussion

Overall, there is insufficient evidence to support vitamin D supplementation for reducing asthma exacerbation, improving asthma control or enhancing lung function in children, while some evidence suggests that the supplementation may reduce asthma recurrence. Although vitamin D supplementation significantly increased serum 25(OH)D concentrations, this biochemical improvement did not translate into consistent clinical benefits. This suggests that correction of vitamin D status alone may be insufficient to modify the complex inflammatory and clinical course of childhood asthma.

The observed reduction in asthma recurrence should be interpreted cautiously and may reflect a more preventive rather than therapeutic role of vitamin D. Clinically, recurrence may refer more broadly to the return of asthma symptoms or episodes after a period of relative stability, whereas outcomes such as exacerbations requiring systemic corticosteroids, emergency department visits, or hospitalization generally represent more acute and severe deteriorations. In addition, recurrence-related outcomes may also have been defined more broadly or less stringently across primary trials and reviews, making them more likely to capture earlier or milder events. Vitamin D supplementation may therefore be more likely to influence susceptibility to recurrent or infection-triggered episodes through immunomodulatory effects and enhanced host defense, while having a limited impact on more severe exacerbations that reflect established airway inflammation, disease severity, and background controller treatment. This difference in clinical meaning and outcome definition may partly explain why a signal was observed for asthma recurrence but not for most other exacerbation endpoints. However, the evidence for asthma recurrence was limited and classified as weak (class IV), and definitions were not standardized across studies (Supplementary Table S6). Therefore, this result should be considered hypothesis-generating evidence of clinical benefit requiring further confirmation.

The absence of a clear benefit should not necessarily be interpreted as definitive evidence of ineffectiveness in all pediatric patients. For example, one subgroup analysis showed a significantly reduced risk of asthma exacerbation among children with baseline serum 25(OH)D levels below 10 ng/mL (RR = 0.48, 95%CI, 0.28 to 0.83) [22]. This finding is biologically plausible, as children with profound vitamin D deficiency may be more likely to benefit from supplementation than those with sufficient levels. However, such high-risk populations were underrepresented in many of the included reviews and primary trials, limiting the robustness and generalizability of subgroup-specific conclusions. Future randomized controlled trials should therefore focus more explicitly on children with severe deficiency, poor asthma control, frequent exacerbations, or more severe asthma phenotypes. Thus, the absence of a consistent overall benefit should not be interpreted as evidence against a possible benefit in specific subgroups, but rather as a reflection of the limitations of the current review-level evidence base.

Several factors may explain the inconsistency of findings across trials and reviews. First, the included RCTs were clinically heterogeneous with respect to participant age, baseline vitamin D status, asthma severity, background therapy, supplementation dose, administration regimen, and follow-up duration. These differences are likely to have influenced treatment responsiveness and contributed to the moderate heterogeneity observed for exacerbation outcomes. Second, outcome definitions were not fully consistent across studies (Supplementary Table S6). Asthma exacerbations were variably defined as overall exacerbation events, exacerbations requiring systemic corticosteroids, or exacerbations leading to emergency department visits or hospitalization. Likewise, asthma control and lung function were measured using different tools and indicators, making direct comparison across studies more difficult. Third, pediatric asthma itself is a heterogeneous disease, and vitamin D may plausibly exert different effects across allergic, infection-prone, or more severe inflammatory phenotypes. Such complexity is not easily captured in pooled analyses based on broad clinical categories.

Another possible explanation is the temporal evolution of both asthma management and vitamin D exposure in the general population [56]. Earlier randomized trials and meta-analyses tended to suggest more favorable effects of vitamin D supplementation [7,35], whereas more recent studies have more often reported null results [10,42]. One possible reason is that background vitamin D intake from diet may have increased over time, thereby reducing between-group contrasts in later trials. In addition, modern asthma care has become more standardized and effective, with broader use of inhaled corticosteroids and other controller medications. Under these circumstances, the incremental benefit of vitamin D supplementation as an adjunctive therapy may be too small to detect unless studied in carefully selected high-risk populations. This may also help explain why vitamin D increased circulating 25(OH)D levels without producing corresponding improvements in clinical endpoints. However, most included trials did not stratify results according to concomitant asthma medications, baseline inflammatory characteristics, or infection-related triggers. As a result, pooled estimates may have obscured clinically relevant interactions between vitamin D supplementation and existing anti-inflammatory therapies.

The certainty and credibility of the evidence further limit confidence in the observed findings. Most outcomes were supported by low or very low certainty evidence, and the methodological quality of the included systematic reviews was often suboptimal. Many meta-analyses were based on a small number of trials with limited sample sizes, increasing vulnerability to imprecision and unstable pooled estimates. In addition, substantial variation in study design, intervention protocols, and outcome measurement reduced comparability across studies. Therefore, the absence of convincing evidence for benefit should be interpreted as reflecting both a likely lack of large, consistent clinical effects and important limitations in the current evidence base. These findings should also be interpreted in the context of current asthma guidance. GINA recognizes that low vitamin D status is associated with worse asthma-related outcomes and notes that supplementation may be beneficial in some patients with low baseline 25(OH)D levels; however, it also states that there is no good-quality evidence that vitamin D supplementation consistently improves asthma control or reduces exacerbations overall [57]. Our findings are therefore broadly aligned with this guidance, rather than contradictory to it.

Another important finding of this umbrella review is that the evidence was extensive in appearance but limited in independence. Although 14 systematic reviews were included, they were based on only 26 unique primary randomized controlled trials, and the CCA indicated very high overlap across reviews. This suggests that the field has generated repeated syntheses of a relatively small and highly overlapping body of evidence. Consequently, future research efforts should shift from conducting yet another systematic review to funding innovative primary studies designed to answer the remaining uncertainties, and promoting data sharing and individual patient data meta-analyses to maximize the value of existing trials. Only by expanding the high-quality evidence base can the field move beyond this cycle of redundancy.

To our knowledge, this is the first umbrella review to comprehensively synthesize evidence on vitamin D supplementation in childhood asthma. In addition, we systematically evaluated methodological quality, certainty of evidence, and credibility of outcomes using AMSTAR 2, the GRADE approach, and evidence classification criteria, providing a transparent and hierarchical assessment of the available evidence. Nevertheless, several limitations should be acknowledged. First, as with all umbrella reviews, our conclusions depend on the quality of the included systematic reviews and the primary RCTs on which they were based. Second, we extracted all eligible meta-analytic estimates, but for evidence grading, we relied on the most comprehensive meta-analysis for each outcome, which may not fully capture all nuances across competing analyses. This pragmatic approach may have favored more recent or larger reviews over methodologically stronger but older reviews. However, most reviews included were rated as low or critically low quality, and the meta-analyses selected for GRADE assessment were also predominantly from the low-quality category when duplicate outcomes were present. Therefore, restricting GRADE assessment to only high- or moderate-quality reviews would not materially alter the overall interpretation of the evidence. Finally, some potentially important subgroup effects, such as those related to baseline vitamin D deficiency, asthma phenotype, or concomitant treatment, could not be robustly examined because of limited and inconsistent reporting in the underlying reviews. Several clinically important modifiers, such as residence-related sun exposure, lifestyle, diet or nutritional status, and social background, were also absent across the included systematic reviews. Therefore, our findings should be interpreted as a synthesis of the current review-level evidence base rather than as a definitive assessment of vitamin D supplementation across all clinically relevant pediatric asthma subgroups. This limitation also underscores an important gap in the current evidence base, highlighting the need for future primary trials and more detailed evidence syntheses that explicitly account for clinically relevant effect modifiers.

## 5. Conclusions

In conclusion, current evidence does not support routine vitamin D supplementation as an effective strategy to improve asthma control, reduce exacerbation, or enhance lung function in children with asthma despite its effect on increasing serum 25-hydroxyvitamin D levels and a possible benefit for asthma recurrence. However, the possibility of benefit in selected high-risk subgroups, particularly children with profound vitamin D deficiency or more severe disease, remains open. Future trials should prioritize clearly defined target populations, standardized supplementation regimens, consistent outcome definitions, and adequate follow-up to determine whether vitamin D has a clinically meaningful role in precision management of childhood asthma.

## Figures and Tables

**Table 1:** Characteristics of the systematic reviews included in this umbrella review.

Author (Year)	Population	Baseline 25(OH)D	*n*, RCTs/ *No.*, Participants	Follow-Up	Dosage	Outcome	Risk of Bias Assessment Tool
Pojsupap 2015 [20]	children with asthma; 5–18 years	13.0–35.6 ng/mL	5/625	4–52 weeks	500–2000 IU/day	asthma exacerbations	Cochrane
Williamson 2023 [21] †	children with asthma; 0–18 years	11.2–35.6 ng/mL	15/1155	3–40 months	400–4000 IU/day	asthma exacerbations	Cochrane
Li 2022 [22]	children with asthma; 1–18 years	11.2–31.7 ng/mL	18/1478	6 weeks–12 months	1000–60,000 IU/week	asthma exacerbations, asthma control, pulmonary function test, serum vitamin D levels	Cochrane
Wang 2022 [23]	children with asthma; 0–18 years	19.0–76.3 ng/mL	12/1871	6 weeks–6 months	200–2000 IU/day	asthma exacerbations, asthma control, pulmonary function test	Jadad
Fares 2015 [24]	children with asthma; 5–18 years	31.3–36.1 ng/mL	4/149	6 weeks–19 months	1000–4200 IU/week	FEV1%, serum vitamin D levels	Cochrane
Riverin 2015 [25]	children with asthma; 3–18 years	-	8/573	1–12 months	1000 IU/week–60,000 IU/month	asthma exacerbations, FEV1%, serum vitamin D levels	Cochrane
Sobczak 2023 [26] †	children with asthma; 4–16 years	10.8–23.2 ng/mL	3/354	3–9 months	1000–2000 IU/day	asthma control, FEV1%	Cochrane
Chen 2021 [27] †	children with asthma; 3–10 years	10.8–36.1 ng/mL	7/705	2–12 months	1000–28,000 IU/week	asthma exacerbations	Cochrane
Liu 2022 [28] †	children with asthma; 3–18 years	10.8–16.5 ng/mL	4/458	1–9 months	1000–2000 IU/day	asthma exacerbations, FEV1%	Cochrane
Kumar 2022 [29]	children with asthma; 1–18 years	10.8–36.1 ng/mL	18/1579	1–12 months	1000–60,000 IU/week	asthma exacerbations, asthma control, pulmonary function test, serum vitamin D levels	Cochrane
Hao 2022 [30]	children with asthma; 3–14 years	10.8–29.4 ng/mL	8/738	6 weeks–12 months	400–4000 IU/day	asthma exacerbations, pulmonary function test, serum vitamin D levels, adverse effects	Cochrane
Fedora 2024 [31]	children with asthma; 0–18 years	10.8–36.1 ng/mL	10/1243	3–12 months	150–4000 IU/day	asthma exacerbations, FEV1%, serum vitamin D levels	JBI
El Abd 2024 [32] †	children with asthma; 6–18 years	15.8–29.0 ng/mL	5/406	6–48 weeks	800–4000 IU/day	serum IgE, blood eosinophils	Cochrane
Wang 2019 [33] †	children with asthma; 1–18 years	20.0–36.1 ng/mL	5/183	6 weeks–12 months	400–2000 IU/day	asthma exacerbations, FEV1%	Cochrane

† Studies also included adults; however, only data for children’s subgroups were extracted. Abbreviations: FEV1%, precent predicted forced expiratory volume in 1 second; IgE, immunoglobulin E in serum; JBI, the Joanne Briggs Institute Critical Appraisal tools.

**Table 2:** Results of the quality assessment of systematic reviews with the AMSTAR 2 Tool.

Reference	1. PICO	2. A Priori Study Protocol	3. Choice Study Design	4. Search Strategy	5. Duplicate Study Selection	6. Duplicate Data Extraction	7. List of Excluded Studies	8. Description Included Studies	9. Technique RoB	10. Funding Sources of Included Studies	11. Statistical Methods	12. Impact of RoB on Results	13. RoB Discussion	14. Heterogeneity Discussion	15. Publication Bias	16. Conflict of Interest	Overall
Pojsupap 2015 [20]	Y	N	Y	PY	Y	Y	N	Y	Y	N	Y	Y	Y	Y	Y	Y	Critically low
Williamson 2023 [21]	Y	PY	N	Y	Y	Y	Y	Y	Y	Y	Y	Y	Y	Y	Y	Y	High
Li 2022 [22]	Y	PY	N	PY	Y	Y	N	PY	Y	N	Y	Y	Y	Y	Y	Y	Low
Wang 2022 [23]	Y	N	N	PY	Y	Y	N	PY	Y	N	Y	N	N	Y	N	Y	Critically low
Fares 2015 [24]	Y	PY	N	PY	Y	Y	N	Y	Y	Y	Y	Y	Y	Y	Y	Y	Low
Riverin 2015 [25]	Y	N	N	PY	Y	Y	Y	Y	PY	N	Y	N	Y	Y	Y	Y	Low
Sobczak 2023 [26]	Y	N	N	PY	N	N	N	PY	Y	N	Y	N	N	Y	Y	Y	Critically low
Chen 2021 [27]	Y	N	N	PY	Y	Y	N	PY	Y	N	Y	Y	Y	Y	Y	Y	Critically low
Liu 2022 [28]	Y	PY	N	PY	Y	Y	N	PY	Y	N	Y	N	N	Y	Y	Y	Critically low
Kumar 2022 [29]	Y	PY	Y	PY	Y	Y	N	Y	Y	N	Y	Y	Y	Y	Y	Y	Low
Hao 2022 [30]	Y	PY	N	PY	Y	Y	N	PY	Y	N	Y	Y	Y	Y	Y	Y	Low
Fedora 2024 [31]	Y	PY	Y	PY	Y	Y	N	PY	PY	N	Y	N	N	N	Y	Y	Critically low
El Abd 2024 [32]	Y	PY	Y	PY	Y	Y	N	PY	Y	N	Y	Y	Y	Y	N	Y	Critically low
Wang 2019 [33]	Y	PY	N	PY	Y	Y	N	PY	Y	N	Y	Y	Y	Y	Y	Y	Low

Abbreviations: Y = yes, N = no, PY = partial yes.

## Data Availability

All data relevant to this study are included in the published article and its Supplementary Materials, which are fully available and accessible.

## Supplementary Materials

The following supporting information can be downloaded at: https://www.mdpi.com/article/10.3390/nu18101560/s1. Methods S1: Selection process for meta-analyses addressing the same outcome; Table S1: Detailed search strategies; Table S2: Classification of evidence in meta-analysis; Table S3: Results of meta-analyses of RCTs on effects of vitamin D supplementation in children with asthma; Table S4: Primary randomized controlled trials included in each systematic review; Table S5: Certainty of evidence of the included meta-analyses of RCTs on effects of vitamin D supplementation in children with asthma by using the GRADE; Table S6: Definitions of key asthma-related outcomes across included reviews.
