# Supplementary material for: Vitamin D Supplementation in Children with Asthma: An Umbrella Review
Source: Nutrients. 2026 May 14;18(10):1560. doi: 10.3390/nu18101560 (PMC13210331; doi:10.3390/nu18101560)
Supplement: Supplementary file 1 [file nutrients-18-01560-s001.zip › nutrients-4191607-supplementary.pdf]

Supplementary Materials

Vitamin D Supplementation in Children with Asthma: An Umbrella Review

Jianzhao Liu †, Yujun Long † and Zhirong Yang*

Contents

Methods S1. Selection process for meta-analyses addressing the same outcome............... 1

Table S1. Detailed search strategies. 2

Table S2. Classification of evidence in meta-analysis.... 10

Table S3. Results of meta-analyses of RCTs on effects of vitamin D supplementation in children with asthma....11

Table S4. Primary randomized controlled trials included in each systematic review. 14

Table S5. Certainty of evidence of the included meta-analyses of RCTs on effects of vitamin D supplementation in children with asthma by using the GRADE. 16

Table S6. Definitions of key asthma-related outcomes across included reviews.............. 19

References 20

Methods S1. Selection process for meta-analyses addressing the same outcome.

1. Identify all eligible meta-analyses for the same outcome.
2. Select the most recent and/or largest review to represent the most comprehensive and updated synthesis.
3. Use AMSTAR 2 to assess the methodological quality of all included reviews.
4. Interpret GRADE findings in conjunction with AMSTAR 2 ratings, particularly where the selected review is of low methodological quality.

Table S1. Detailed search strategies.

PubMed

1	"vitamin d"[MeSH Terms] OR "ergocalciferols"[MeSH Terms]
2	"vitamin d deficiency"[MeSH Terms]
3	"vitamin d*"[Text Word]
4	"alfacalcidol*"[Text Word]
5	"alpha calcidol*"[Text Word]
6	"calciol"[Text Word]
7	"calcidiol"[Text Word]
8	"calcifediol*"[Text Word]
9	"calciferol"[Text Word]
10	"calcipotriol"[Text Word]
11	"calcitriol*"[Text Word]
12	"calderol"[Text Word]
13	"cholecalciferol*"[Text Word]
14	"colecalciferol*"[Text Word]
15	"dedrogyl"[Text Word]
16	"dihydrotachysterol*"[Text Word]
17	"dihydroxycolecalciferol"[Text Word]
18	"dihydroxyvitamin d*"[Text Word]
19	"doxercalciferol"[Text Word]
20	"eldecalcitol"[Text Word]
21	"epicalcitriol"[Text Word]
22	"ercalcidiol"[Text Word]
23	"ergocalciferol*"[Text Word]
24	"falecalcitriol"[Text Word]
25	"hidroferol"[Text Word]
26	"hydroxyvitamin d*"[Text Word]
27	"hydroxycalciferol"[Text Word]
28	"Hydroxycholecalciferols"[Text Word]
29	"hydroxyergocalciferol*"[Text Word]
30	"lexicalcitol"[Text Word]
31	"maxacalcitol"[Text Word]
32	"oxacalcitriol"[Text Word]
33	"paricalcitol"[Text Word]
34	"seocalcitol"[Text Word]
35	"tachystin"[Text Word]
36	"25 oh d"[Text Word]
37	"vitamin d"[MeSH Terms] OR "ergocalciferols"[MeSH Terms] OR "vitamin d deficiency"[MeSH Terms] OR "vitamin d*"[Text Word] OR "alfacalcidol*"[Text Word] OR "alpha calcidol*"[Text Word] OR "calciol"[Text Word] OR "calcidiol"[Text Word] OR "calcifediol*"[Text Word] OR "calciferol"[Text Word] OR "calcipotriol"[Text Word] OR

"calcitriol*"[Text Word] OR "calderol"[Text Word] OR "cholecalciferol*"[Text Word] OR "colecalciferol*"[Text Word] OR "dedrogyl"[Text Word] OR "dihydrotachysterol*"[Text Word] OR "dihydroxycolecalciferol"[Text Word] OR "dihydroxyvitamin d*"[Text Word] OR "doxercalciferol"[Text Word] OR "eldecalcitol"[Text Word] OR "epicalcitriol"[Text Word] OR "ercalcidiol"[Text Word] OR "ergocalciferol*"[Text Word] OR "falecalcitriol"[Text Word] OR "hidroferol"[Text Word] OR "hydroxyvitamin d*"[Text Word] OR "hydroxycalciferol"[Text Word] OR "Hydroxycholecalciferols"[Text Word] OR "hydroxyergocalciferol*"[Text Word] OR "lexicalcitol"[Text Word] OR "maxacalcitol"[Text Word] OR "oxacalcitriol"[Text Word] OR "paricalcitol"[Text Word] OR "seocalcitol"[Text Word] OR "tachystin"[Text Word] OR ((25[UID] AND ("hydroxide ion"[Supplementary Concept] OR "hydroxide ion"[All Fields] OR "oh"[All Fields])) AND "D"[Text Word])

38	"randomized controlled trial"[Publication Type]
39	"controlled clinical trial"[Publication Type]
40	"randomized"[Title/Abstract]
41	"placebo"[Title/Abstract]
42	"clinical trials as topic"[MeSH Terms]
43	"randomly"[Title/Abstract]
44	"trial"[Title]
45	"randomized controlled trial"[Publication Type] OR "controlled clinical trial"[Publication Type] OR "randomized"[Title/Abstract] OR "placebo"[Title/Abstract] OR "clinical trials as topic"[MeSH Terms] OR "randomly"[Title/Abstract] OR "trial"[Title]
46	("animals"[MeSH Terms:noexp] OR "animals"[All Fields]) NOT "humans"[MeSH Terms]
47	("randomized controlled trial"[Publication Type] OR "controlled clinical trial"[Publication Type] OR "randomized"[Title/Abstract] OR "placebo"[Title/Abstract] OR "clinical trials as topic"[MeSH Terms] OR "randomly"[Title/Abstract] OR "trial"[Title]) NOT (("animals"[MeSH Terms:noexp] OR "animals"[All Fields]) NOT "humans"[MeSH Terms])
48	("vitamin d"[MeSH Terms] OR "ergocalciferols"[MeSH Terms] OR "vitamin d deficiency"[MeSH Terms] OR "vitamin d*"[Text Word] OR "alfacalcidol*"[Text Word] OR "alpha calcidol*"[Text Word] OR "calciol"[Text Word] OR "calcidiol"[Text Word] OR "calcifediol*"[Text Word] OR "calciferol"[Text Word] OR "calcipotriol"[Text Word] OR "calcitriol*"[Text Word] OR "calderol"[Text Word] OR "cholecalciferol*"[Text Word] OR "colecalciferol*"[Text Word] OR "dedrogyl"[Text Word] OR "dihydrotachysterol*"[Text Word] OR "dihydroxycolecalciferol"[Text Word] OR "dihydroxyvitamin d*"[Text Word] OR "doxercalciferol"[Text Word] OR "eldecalcitol"[Text Word] OR "epicalcitriol"[Text Word] OR "ercalcidiol"[Text Word] OR "ergocalciferol*"[Text Word] OR "falecalcitriol"[Text Word] OR "hidroferol"[Text Word] OR "hydroxyvitamin d*"[Text Word] OR "hydroxycalciferol"[Text Word] OR "Hydroxycholecalciferols"[Text Word] OR "hydroxyergocalciferol*"[Text Word] OR "lexicalcitol"[Text Word] OR "maxacalcitol"[Text Word] OR "oxacalcitriol"[Text Word] OR "paricalcitol"[Text Word] OR "seocalcitol"[Text Word] OR "tachystin"[Text Word] OR ((25[UID] AND ("hydroxide ion"[Supplementary Concept] OR "hydroxide ion"[All Fields] OR "oh"[All Fields])) AND

"D"[Text Word])) AND (("randomized controlled trial"[Publication Type] OR "controlled clinical trial"[Publication Type] OR "randomized"[Title/Abstract] OR "placebo"[Title/Abstract] OR "clinical trials as topic"[MeSH Terms] OR "randomly"[Title/Abstract] OR "trial"[Title]) NOT (("animals"[MeSH Terms:noexp] OR "animals"[All Fields]) NOT "humans"[MeSH Terms]))

49 Meta-Analysis as Topic/
50 meta analy$.tw.
51 metaanaly$.tw.
52 Meta-Analysis/
53 (systematic adj (review$1 or overview$1)).tw.
54 exp Review Literature as Topic/
55 or/49-54
56 cochrane.ab.
57 embase.ab.
58 (psychlit or psyclit).ab.
59 (psychinfo or psycinfo).ab.
60 (cinahl or cinhal).ab.
61 science citation index.ab.
62 bids.ab.
63 cancerlit.ab.
64 or/56-63
65 reference list$.ab.
66 bibliograph$.ab.
67 hand-search$.ab.
68 relevant journals.ab.
69 manual search$.ab.
70 or/65-69
71 selection criteria.ab.
72 data extraction.ab.
73 71 or 72
74 Review/
75 73 and 74
76 Comment/
77 Letter/
78 Editorial/
79 animal/
80 human/
81 79 not (79 and 80)
82 or/76-78,81
83 55 or 64 or 70 or 75
84 83 not 82
85 48 and 84

Embase

#1. 'vitamin d'/exp OR 'vitamin d'
#2. 'vitamin d deficiency'/exp OR 'vitamin d deficiency'
#3. 'vitamin d*'
#4. 'alfacalcidol*'
#5. 'alphacalcidol*'
#6. 'calcidiol'
#7. 'calcifediol*'
#8. 'calciferol'
#9. 'calcitriol*'
#10. 'calcipotriol'
#11. 'cholecalciferol*'
#12. 'colecalciferol*'
#13. 'doxercalciferol'
#14. 'dihydrotachysterol*'
#15. 'dihydroxyvitamin'
#16. 'ergocalciferol*'
#17. 'epicalcitriol'
#18. 'falecalcitriol'
#19. 'hydroxyvitamin d*'
#20. 'hydroxycolecalciferol'
#21. 'lexacalcitol'
#22. 'maxicalcitol'
#23. 'oxacalcitriol'
#24. 'paricalcitol'
#25. 'seocalcitol'
#26. 'tacalcitol'
#27. '25(oh)d'
#28. #1 OR #2 OR #3 OR #4 OR #5 OR #6 OR #7 OR #8 OR #9 OR #10 OR #11 OR #12 OR #13 OR #14 OR #15 OR #16 OR #17 OR #18 OR #19 OR #20 OR #21 OR #22 OR #23 OR #24 OR #25 OR #26 OR #27
#29. 'randomized controlled trial'
#30. 'controlled clinical study'
#31. 'random$':ab,ti
#32. 'randomization'
#33. 'intermethod comparison'
#34. 'placebo':ab,ti
#35. compare:ti OR compared:ti OR comparison:ti
#36. (evaluated:ab OR evaluate:ab OR evaluating:ab OR assessed:ab OR assess:ab) AND (compare:ab OR compared:ab OR comparing:ab OR comparison:ab)
#37. open:ab,ti AND label:ab,ti
#38. (double:ab,ti OR single:ab,ti OR doubly:ab,ti OR singly:ab,ti) AND (blind:ab,ti OR blinded:ab,ti OR blindly:ab,ti)

#39. 'double blind procedure'
#40. 'parallel group$':ab,ti
#41. crossover:ab,ti OR 'cross over':ab,ti
#42. (assign$:ab,ti OR match:ab,ti OR matched:ab,ti OR allocation:ab,ti) AND (alternate:ab,ti OR group$:ab,ti OR intervention$:ab,ti OR patient$:ab,ti OR subject$:ab,ti OR participant$:ab,ti)
#43. assigned:ab,ti OR allocated:ab,ti
#44. controlled:ab,ti AND (study:ab,ti OR design:ab,ti OR trial:ab,ti)
#45. volunteer:ab,ti OR volunteers:ab,ti
#46. 'human experiment'
#47. 'trial':ti
#48. #29 OR #30 OR #31 OR #32 OR #33 OR #34 OR #35 OR #36 OR #37 OR #38 OR #39 OR #40 OR #41 OR #42 OR #43 OR #44 OR #45 OR #46 OR #47
#49. random$:ab,ti AND sampl$:ab,ti AND ("cross section$":ab,ti OR questionnaire$:ab,ti OR survey$:ab,ti OR database$:ab,ti)
#50. 'comparative study/':ab,ti OR 'controlled study/':ab,ti OR 'randomi?ed controlled.ti,ab.':ab,ti OR 'randomly assigned.ti,ab.':ab,ti
#51. #49 NOT #50
#52. 'cross-sectional study/':ab,ti NOT ('randomized controlled trial/':ab,ti OR 'controlled clinical study/':ab,ti OR 'controlled study/':ab,ti OR 'randomi?ed controlled.ti,ab.':ab,ti OR 'control group$1.ti,ab.':ab,ti)
#53. case:ab,ti AND control$:ab,ti AND random$:ab,ti NOT 'randomi?ed controlled':ab,ti
#54. 'systematic review':ti NOT (trial:ti OR study:ti)
#55. nonrandom$:ab,ti NOT random$:ab,ti
#56. "random field$":ab,ti
#57. 'random cluster':ab,ti AND sampl$:ab,ti
#58. review:ab
#59. review:it
#60. #58 AND #59
#61. trial:ti
#62. #60 NOT #61
#63. 'we searched':ab
#64. review:ti
#65. #59 OR #64
#66. #63 AND #65
#67. 'update review':ab
#68. databases:ab AND searched:ab
#69. rat:ti OR rats:ti OR mouse:ti OR mice:ti OR swine:ti OR porcine:ti OR murine:ti OR sheep:ti OR lambs:ti OR pigs:ti OR piglets:ti OR rabbit:ti OR rabbits:ti OR cat:ti OR cats:ti OR dog:ti OR dogs:ti OR cattle:ti OR bovine:ti OR monkey:ti OR monkeys:ti OR trout:ti OR marmoset$:ti
#70. '(animal experiment)'
#71. #69 AND #70

#72. 'animal experiment' NOT ('human experiment' OR human)
#73. #49 OR #50 OR #51 OR #52 OR #53 OR #54 OR #55 OR #62 OR #66 OR #67 OR #68 OR #71 OR #72
#74. #48 NOT #73
#75. #28 AND #74
#76. exp Meta Analysis/
#77. ((meta adj analy$) or metaanalys$).tw.
#78. (systematic adj (review$1 or overview$1)).tw.
#79. or/76-78
#80. cancerlit.ab.
#81. cochrane.ab.
#82. embase.ab.
#83. (psychlit or psyclit).ab.
#84. (psychinfo or psycinfo).ab.
#85. (cinahl or cinhal).ab.
#86. science citation index.ab.
#87. bids.ab.
#88. or/80-87
#89. reference lists.ab.
#90. bibliograph$.ab.
#91. hand-search$.ab.
#92. manual search$.ab.
#93. relevant journals.ab.
#94. or/89-93
#95. data extraction.ab.
#96. selection criteria.ab.
#97. 95 or 96
#98. review.pt.
#99. 97 and 98
#100. letter.pt.
#101. editorial.pt.
#102. animal/
#103. human/
#104. 102 not (102 and 103)
#105. or/100-101,104
#106. 79 or 88 or 94 or 99
#107. 106 not 105
#108. 75 and 107

The Cochrane Library

#1 MeSH descriptor: [Vitamin D] explode all trees
#2 (Vitamin D):ti,ab,kw
#3 (Vitamin D deficiency):ti,ab,kw
#4 (Vitamin D*):ti,ab,kw
#5 (alfacalcidol*):ti,ab,kw
#6 (alpha-calcidol*):ti,ab,kw
#7 (calcidiol):ti,ab,kw
#8 (calcifediol*):ti,ab,kw
#9 (calciferol*):ti,ab,kw
#10 (calciol):ti,ab,kw
#11 (calcipotriol):ti,ab,kw
#12 (calcitriol*):ti,ab,kw
#13 (cholecalciferol*):ti,ab,kw
#14 (colecalciferol*):ti,ab,kw
#15 (dihydrotachysterol*):ti,ab,kw
#16 (dihydroxycolecalciferol):ti,ab,kw
#17 (dihydroxycholecalciferol):ti,ab,kw
#18 (dihydroxyvitamin D*):ti,ab,kw
#19 (doxercalciferol):ti,ab,kw
#20 (eldecalcitol):ti,ab,kw
#21 (ergocalciferol*):ti,ab,kw
#22 (falecalcitriol):ti,ab,kw
#23 (hidroferol):ti,ab,kw
#24 (hydroxyvitamin D*):ti,ab,kw
#25 (hydroxycholecalciferols):ti,ab,kw
#26 (hydroxycolecalciferol):ti,ab,kw
#27 (lexacalcitol):ti,ab,kw
#28 (oxacalcitriol):ti,ab,kw
#29 (paricalcitol):ti,ab,kw
#30 (seocalcitol):ti,ab,kw
#31 (tacalcitol):ti,ab,kw
#32 or/1-31
#33 randomized controlled trial
#34 controlled clinical trial
#35 (randomized):ti,ab,kw
#36 (placebo):ti,ab,kw
#37 MeSH descriptor: [Clinical Trials as Topic] this term only
#38 (randomly):ti,ab,kw
#39 (trial):ti
#40 #33or#34or#35or#36or#37or#38or#39
#41 animals
#42 humans

#43 #41 not #42
#44 #40 not #43
#45 #32and #44
#46 systematic* [ti] and review [ti]
#47 Systematic overview* [ti] or Cochrane review* [ti] or systemic review* [ti] or scoping review [ti] or scoping literature review [ti] or mapping review [ti] or Umbrella review* [ti] or (review of reviews [ti] or overview of reviews [ti]) or meta-review [ti] or (integrative review [ti] or integrated review [ti] or integrative overview [ti] or meta-synthesis [ti] or metasynthesis [ti] or quantitative review [ti] or quantitative synthesis [ti] or research synthesis [ti] or meta-ethnography [ti]) or Systematic literature search [ti] or Systematic literature research [ti] or meta-analyses [ti] or metaanalyses [ti] or metaanalysis [ti] or meta-analysis [ti] or meta-analytic review [ti] or meta-analytical review [ti] or meta-analysis [pt]
#48 search* [tiab] or medline [tiab] or pubmed [tiab] or embase [tiab] or Cochrane [tiab] or scopus [tiab] or web of science [tiab] or sources of information [tiab] or data sources [tiab] or following databases [tiab]
#49 study selection [tiab] or selection criteria [tiab] or eligibility criteria [tiab] or inclusion criteria [tiab] or exclusion criteria [tiab]
#50 #48 and #49
#51 #46 or #47 or #50 or systematic review [pt]
#52 letter [pt] or editorial [pt] or comment [pt] or case reports [pt] or historical article [pt] or report [ti] or protocol [ti] or protocols [ti] or withdrawn [ti] or retraction of publication [pt] or retraction of publication as topic [mesh] or retracted publication [pt] or reply [ti] or published erratum [pt]
#53 #51 not #52
#54 #45 and #53

Table S2. Classification of evidence in meta-analysis.

**Convincing evidence (class I)**
≥1000 cases
p≤10^-6 for random effect models
Low to moderate between-studies heterogeneity (I^2≤50%)
95% CIs excluding the null value
No evidence of publication bias

**Highly suggestive evidence (class II)**
≥1000 cases
p≤10^-6 for random effect models
Class 1 was not met

**Suggestive evidence (class III)**
≥1000 cases
p≤10^-3 for random effect models
Class 2 was not met

**Weak evidence (class IV)**
p≤0·05

**Non-significant evidence (class V)**
p>0·05

Table S3. Results of meta-analyses of RCTs on effects of vitamin D supplementation in children with asthma.

Outcome	Reference	RCTs, n	Participants, (I, C)	Effect measure	Pooled effect size (95% CI)	Unit	*P* value	I² (%)	Credibility
**asthma exacerbations**									
	Li 2022	11	1143（577/566）	RR	0.84 (0.65, 1.08)	-	0.18	59	Class V
	Pojsupap 2015	3	482（241/241）	RR	0.41 (0.27, 0.63)	-	<0.0001	0	Class IV
	Riverin 2015	3	482（241/242）	RR	0.41 (0.27, 0.63)	-	<0.0001	0	Class IV
	Chen 2021	4	387（193/194）	RR	0.69 (0.55, 0.87)	-	0.001	0	Class IV
	Liu 2022	3	204（102/102）	RR	0.46 (0.30, 0.70)	-	-	0	Class IV
	Kumar 2022	11	1132	RR	0.84 (0.65, 1.09)	-	-	58	Class V
	Hao 2022	4	353（185/168）	RR	0.92 (0.68, 1.25)	-	0.6	1	Class V
	Fedora 2024	8	1169	RR	0.62 (0.44, 0.87)	-	0.006	61	Class IV
	Wang 2019	2	70（35/35）	RR	0.61 (0.24, 1.58)	-	0.31	65	Class V
**asthma recurrence rate**									
	Wang 2022	6	802（410/392）	RR	0.53 (0.35, 0.79）	-	0.002	16	Class IV
**asthma exacerbations requiring systemic corticosteroids**									
	Williamson 2023	9	837（422/415）	OR	1.28 (0.83, 1.97）	-	0.26	0	Class V
	Li 2022	5	253（133/120）	RR	0.99 (0.68, 1.46)	-	0.98	0	Class V
	Kumar 2022	6	445（229/216）	RR	1.13 (0.86, 1.48)	-	0.38	0	Class V
	Hao 2022	4	231（122/109）	RR	1.03 (0.41, 2.57	-	0.95	56	Class V
**requiring emergency department visit or hospitalization, or both**									
	Li 2022	5	445（232/213）	RR	1.01 (0.91, 1.12)	-	0.82	0	Class V
**requiring emergency/unscheduled visits**									
	Kumar 2022	3	361	RR	0.97 (0.89, 1.07)	-	-	0	Class V
	Hao 2022	3	125（62/63）	RR	1.13 (0.77, 1.65)	-	0.53	7	Class V
**requiring hospitalisations for asthma exacerbation**									

Kumar 2022	2	275（146/129）	RR	1.38 (0.52, 3.66)	-	-	0	Class V
Hao 2022	4	359（189/170）	RR	1.20 (0.48, 2.96)	-	0.7	0	Class V
**childhood asthma control test (C-ACT) score**								
Hao 2022	3	315（157/158）	MD	0.15 (-0.43, 0.74)	-	0.61	0	Class V
**change in C-ACT score**								
Li 2022	4	403（211/192）	MD	-0.34 (-1.92, 1.25)	-	0.68	86	Class V
Sobczak 2023	3	315（157/158）	MD	-0.47 (-2.38, 1.43)	-	<0.01	99	Class IV
**asthma control (no recurrence of asthma within half a year)**								
Wang 2022	4	1041（520/521）	RR	1.01 (0.91, 1.13）	-	0.826	0	Class V
Kumar 2022	4	442	RR	1.00 (0.97, 1.04)	-	-	0	Class V
**change in total asthma symptom (TAS) score**								
Li 2022	2	66（34/32）	MD	0.23 (-0.34, 0.79)	-	0.44	75	Class V
**forced expiratory volume in one second percent predicted (FEV1%)**								
Fares 2015	2	82（41/41）	MD	-0.54 (-5.28, 4.19)	%	0.82	54	Class V
Kumar 2022	4	314	MD	-2.64 (-7.04, 1.77)	%	-	62	Class V
Hao 2022	3	269（135/134）	MD	-4.77 (-9.35, -0.19)	-	0.04	0	Class IV
Wang 2022	5	244（130/114）	SMD	0.49 (-0.05, 1.04）	-	0.078	75.5	Class V
Liu 2022	3	278（142/136）	SMD	-0.30 (-0.54, -0.07)	-	-	0	Class IV
Fedora 2024	4	318（156/162）	SMD	-0.23 (-0.46, -0.01)	-	0.04	5	Class IV
**change in FEV1% predicted**								
Li 2022	7	397（202/195）	MD	0.03 (-3.22, 3.27)	-	0.99	55	Class V
Riverin 2015	4	134（68/66）	MD	0.00 (-3.17, 3.18)	%	1	0	Class V
Sobczak 2023	3	254（129/125）	MD	-3.84 (-7.68, -0.01)	%	<0.01	95	Class IV
Wang 2019	3	98（48/50）	MD	-2.60 (-7.26, 2.06)	%	0.27	63	Class V
**forced vital capacity percent (FVC%)**								
Wang 2022	2	128（71/57）	SMD	0.23 (-0.24, 0.69)	-	0.334	35	Class V
Hao 2022	2	213（107/106）	MD	-5.01 (-9.99, -0.02)	-	0.05	0	Class IV
**FEV1/FVC ratio**								

	Hao 2022	2	213（107/106）	MD	-0.86 (-3.52, 1.79)	-	0.52	0	Class V
**change in FEV1/FVC ratio**									
	Li 2022	3	263（134/129）	MD	-0.02 (-3.03, 3.00)	-	0.99	68	Class V
**change in forced expiratory flow between 25% and 75% of vital capacity (ΔFEF25-75%)**									
	Li 2022	2	191（101/90）	MD	-1.12 (-9.09, 6.85）	-	0.78	0	Class V
**fractional exhaled nitric oxide(FeNO)**									
	Kumar 2022	2	94	MD	-2.87 (-24.66, 18.91)	-	-	/	Class V
	Hao 2022	2	95（18/47）	MD	-3.95 (-22.87, 14.97)	-	0.68	68	Class V
**change in FeNO**									
	Li 2022	2	94（47/47）	MD	7.78 (-1.26, 16.81)	-	0.99	0	Class V
**serum 25(OH)D**									
	Fares 2015	3	116（58/58）	MD	6.56 (-0.64, 13.77)	ng/ml	0.07	97	Class V
	Riverin 2015	5	167（84/83）	MD	19.66 (5.96, 33.37)	nmol/L	0.005	94	Class IV
	Kumar 2022	8	875	MD	10.68 (6.3, 15.05)	ng/ml	-	/	Class IV
	Hao 2022	6	534（262/272）	MD	13.51 (4.24, 22.79)	ng/ml	0.004	98	Class IV
	Fedora 2024	6	542（270/272）	SMD	3.38 (1.66, 5.10)	-	0.0001	98	Class IV
**change in serum 25(OH)D concentration before and after intervention**									
	Li 2022	14	1082（548/534）	MD	11.24 (7.00, 15.47)	ng/mL	<0.00001	93	Class III
**serum total immunoglobulin E (IgE)**									
	EI Abd 2024	2	212（103/109）	MD	0.10 (-0.11, 0.30)	IU/mL	0.36	0	Class V
**change in total IgE**									
	Li 2022	3	244（120/124）	SMD	-0.06 (-0.31, 0.19)	-	0.64	0	Class V
**blood eosinophils**									
	EI Abd 2024	1	38（19/19）	MD	0.01 (-0.13, 0.15)	$10^3/\mu L$	0.89	NA	Class V
**change in interleukin 10 (IL-10)**									
	Li 2022	4	150（73/77）	MD	-6.57 (-27.15, 14.01)	-	0.53	90	Class V
**hypercalciuria**									
	Hao 2022	3	125（62/63）	RR	0.58 (0.22, 1.53）	-	0.27	0	Class V

Table S4. Primary randomized controlled trials included in each systematic review.

SR<br>RCT	Fares 2015[1]	Pojsupap 2015[2]	Riverin 2015[3]	Sobczak 2023[4]	Chen 2021[5]	Liu 2022[6]	Kumar 2022[7]	Hao 2022[8]	Fedora 2024[9]	EI Abd 2024[10]	Williamson 2023[11]	Li 2022[12]	Wang 2022[13]	Wang 2019[14]
Schou 2003[15]	√	√	√										√	
Urashima 2010[16]		√	√				√		√		√	√	√	
Majak 2011[17]	√	√	√		√	√	√		√		√	√	√	√
Lewis 2012[18]	√	√	√				√				√	√		
Yadav 2013[19]		√	√		√	√	√		√		√	√	√	
Darabi 2013[20]			√				√							
Majak 2009[21]	√		√		√		√		√		√	√	√	√
Baris 2014[22]			√		√		√					√	√	
Jat 2021[23]				√	√	√	√	√	√		√	√		
Thakur 2021[24]				√		√	√	√	√	√	√	√		
Kerley 2016[25]				√			√	√		√	√	√	√	√
Ducharme 2019[26]					√		√	√			√	√		
Forno 2020[27]					√		√	√	√		√	√		
Alansari 2017[28]							√					√		
Jensen 2016[29]							√	√			√	√	√	√
Tachimoto 2016[30]							√	√	√	√	√	√	√	
Najmuddin 2017[31]							√					√		
Bar-Yoseph 2015[32]							√	√	√	√		√	√	√
Swangtrakul 2022[33]							√					√		
Kang 2018[34]									√				√	
Rosser 2022[35]										√				
Aglipay 2019[36]											√			
Jerzynska 2016[37]											√			

Chen 2017[38]	√		
El-korashi 2021[39]		√	
Litonjua 2020[40]			√

Table S5. Certainty of evidence of the included meta-analyses of RCTs on effects of vitamin D supplementation in children with asthma by using the GRADE.

Certainty assessment							№ of patients		Effect (95% CI)		Certainty
**№ of studies**	**Study design**	**Risk of bias**	**Inconsistency**	**Indirectness**	**Imprecision**	**Other considerations**	**Intervention**	**Comparison**			
**asthma exacerbations**											
11	randomised trials	serious[a]	serious[b]	not serious	not serious	none	224/577 (38.8%)	250/566 (44.2%)	**RR 0.84** (0.65 to 1.08)	**71 fewer per 1,000** (from 155 fewer to 35 more)	⊕⊕◯◯ Low[a,b]
**asthma recurrence rate**											
6	randomised trials	not serious	not serious	not serious	serious[c]	none	410	392	**RR 0.53** (0.35 to 0.79)		⊕⊕⊕◯ Moderate[c]
**asthma exacerbations requiring systemic corticosteroids**											
9	randomised trials	not serious	not serious	not serious	serious[c]	none	422	415	**OR 1.28** (0.83 to 1.97)		⊕⊕⊕◯ Moderate[c]
**requiring emergency department visit or hospitalization, or both**											
5	randomised trials	not serious	not serious	not serious	serious[c]	none	116/232 (50.0%)	112/213 (52.6%)	**RR 1.01** (0.91 to 1.12)	**5 more per 1,000** (from 47 fewer to 63 more)	⊕⊕⊕◯ Moderate[c]
**childhood asthma control test (C-ACT) score**											
3	randomised trials	not serious	not serious	not serious	serious[c]	none	157	158	MD **0.15 higher** (0.43 lower to 0.74 higher)		⊕⊕⊕◯ Moderate[c]
**asthma control (no recurrence of asthma within half a year)**											

Certainty assessment							№ of patients		Effect (95% CI)	Certainty
№ of studies	Study design	Risk of bias	Inconsistency	Indirectness	Imprecision	Other considerations	Intervention	Comparison		
3	randomised trials	not serious	not serious	not serious	not serious	none	520	521	**RR 1.01** (0.91 to 1.13)	⊕⊕⊕⊕ High
**forced expiratory volume in 1 second percent predicted (FEV1%)**										
5	randomised trials	not serious	serious[b]	not serious	serious[c]	none	130	114	SMD **0.49 SD higher** (0.05 lower to 1.04 higher)	⊕⊕◯◯ Low[b,c]
**forced vital capacity percent (FVC%)**										
2	randomised trials	not serious	not serious	not serious	serious[c]	none	107	106	MD **5.01 lower** (9.99 lower to 0.02 lower)	⊕⊕⊕◯ Moderate[c]
**FEV1/FVC ratio**										
2	randomised trials	not serious	not serious	not serious	serious[c]	none	107	106	MD **0.86 lower** (3.52 lower to 1.79 higher)	⊕⊕⊕◯ Moderate[c]
**fractional exhaled nitric oxide (FeNO)**										
2	randomised trials	not serious	serious[b]	not serious	very serious[d]	none	48	47	MD **3.95 lower** (22.87 lower to 14.97 higher)	⊕◯◯◯ Very low[b,d]
**serum 25(OH)D concentration**										
8	randomised trials	not serious	very serious[e]	not serious	serious[c]	none	875		MD **10.68 higher** (6.3 higher to 15.05 higher)	⊕◯◯◯ Very low[c,e]
**serum total immunoglobulin E (IgE)**										

Certainty assessment							№ of patients		Effect (95% CI)	Certainty
№ of studies	Study design	Risk of bias	Inconsistency	Indirectness	Imprecision	Other considerations	Intervention	Comparison		
2	randomised trials	serious[a]	not serious	not serious	serious[c]	none	103	109	MD **0.1 higher** (0.11 lower to 0.3 higher)	⨁⨁◯◯ Low[a,c]

CI: confidence interval; **MD:** mean difference; **RR:** risk ratio; **OR:** odds ratio; **SMD:** standardised mean difference

Explanations

a. Downgraded by one level for risk of bias: 25-50% of primary studies rated as high risk of bias.

b. Downgraded by one level for inconsistency: I2 was high, and 95% CIs slightly overlap.

c. Downgraded by one level for imprecision: A small number of participants.

d. Downgraded by two levels for imprecision: The number of participants less than 100.

e. Downgraded by two levels for inconsistency: Test for statistical heterogeneity was significant ($p<0.05$) and I2 was very high, and 95% CIs minimally overlap.

Table S6. Definitions of key asthma-related outcomes across included reviews.

Definitions of key asthma-related outcomes were variably reported across included reviews. Some reviews provided explicit definitions for asthma exacerbation, whereas others did not clearly define certain outcomes such as asthma recurrence rate or asthma control. In addition, the operational criteria for asthma exacerbation differed substantially across reviews, ranging from increased use of short-acting β2-agonists to more severe events requiring systemic corticosteroids, emergency department visits, or hospitalization. For outcomes without explicit review-level definitions, definitions were traced back to the original randomized controlled trials when possible.

Reference	Outcome	Reported definition
Li 2022[12]	Asthma exacerbation	At least one of the following: use of systemic corticosteroids for asthma exacerbation; asthma-related emergency department visit or hospitalization; use of short-acting β-agonists as quick-relief medication; or physician-diagnosed exacerbation.
Pojsupap 2015[2]	Asthma exacerbation	Use of short-acting β2-agonists.
Riverin 2015[3]	Asthma exacerbation	Requiring β2-agonists or physician-diagnosed exacerbation.
Liu 2022[6]	Asthma exacerbation	Requiring corticosteroid use or inpatient admission or emergency room visit.
Hao 2022[8]	Asthma exacerbation	Increased symptoms of shortness of breath, cough, wheezing, or chest tightness, with progressive decline in lung function or need for a change in treatment.
Fedora 2024[9]	Asthma exacerbation	Increase in symptoms (shortness of breath, cough, wheezing), progressive decrease in pulmonary function, use of systemic corticosteroids (tablets, suspension, or injection), or increased use of short-acting β2-agonists (SABA).
Wang 2019[14]	Asthma exacerbation	Increase in symptoms of shortness of breath, cough, wheezing, or chest tightness and progressive decrease in pulmonary function, or need for a change in treatment (including short-acting β2-agonists, antibiotics, or oral corticosteroids).
Wang 2022[13]	Asthma recurrence rate	Not reported.
	Asthma control	Not reported.
Kang 2018[34] (primary RCT cited in Wang 2022)	Asthma control rate	No recurrence of asthma within half a year.
	Asthma recurrence rate	At least one acute attack within six months.

References

[1] Fares M M, Alkhaled L H, Mroueh S M, et al. Vitamin D supplementation in children with asthma: a systematic review and meta-analysis[J]. BMC Research Notes, 2015, 8(1): 23.

[2] Pojsupap S, Iliriani K, Sampaio T Z A L, et al. Efficacy of high-dose vitamin D in pediatric asthma: a systematic review and meta-analysis[J]. Journal of Asthma, 2015, 52(4): 382-390.

[3] Riverin B D, Maguire J L, Li P. Vitamin D Supplementation for Childhood Asthma: A Systematic Review and Meta-Analysis[J]. PLOS ONE, 2015, 10(8): e0136841.

[4] Sobczak M, Pawliczak R. Relationship between vitamin D and asthma from gestational to adulthood period: a meta-analysis of randomized clinical trials[J]. BMC Pulmonary Medicine, 2023, 23(1): 212.

[5] Chen Z, Peng C, Mei J, et al. Vitamin D can safely reduce asthma exacerbations among corticosteroid-using children and adults with asthma: a systematic review and meta-analysis of randomized controlled trials[J]. Nutrition Research, 2021, 92: 49-61.

[6] Liu M, Wang J, Sun X. A Meta-Analysis on Vitamin D Supplementation and Asthma Treatment[J]. Frontiers in Nutrition, 2022, 9.

[7] Kumar J, Kumar P, Goyal J P, et al. Vitamin D supplementation in childhood asthma: a systematic review and meta-analysis of randomised controlled trials[J]. ERJ Open Research, 2022, 8(1).

[8] Hao M, Xu R, Luo N, et al. The Effect of Vitamin D Supplementation in Children With Asthma: A Meta-Analysis[J]. Frontiers in Pediatrics, 2022, 10.

[9] Fedora K, Setyoningrum R A, Aina Q, et al. Vitamin D supplementation decrease asthma exacerbations in children: a systematic review and meta-analysis of randomized controlled trials[J]. Annals of Medicine, 2024, 56(1): 2400313.

[10] El Abd A, Dasari H, Dodin P, et al. The effects of vitamin D supplementation on inflammatory biomarkers in patients with asthma: a systematic review and meta-analysis of randomized controlled trials[J]. Frontiers in Immunology, 2024, 15.

[11] Williamson A, Martineau A R, Sheikh A, et al. Vitamin D for the management of asthma[J]. Cochrane Database of Systematic Reviews, 2023, 2023(2).

[12] Li Q, Zhou Q, Zhang G, et al. Vitamin D Supplementation and Allergic Diseases during Childhood: A Systematic Review and Meta-Analysis[J]. Nutrients, 2022, 14(19).

[13] Wang Q, Ying Q, Zhu W, et al. Vitamin D and asthma occurrence in children: A systematic review and meta-analysis[J]. Journal of Pediatric Nursing, 2022, 62: e60-e68.

[14] Wang M, Liu M, Wang C, et al. Association between vitamin D status and asthma control: A meta-analysis of randomized trials[J]. Respiratory Medicine, 2019, 150: 85-94.

[15] Schou A J, Heuck C, Wolthers O D. Does vitamin D administered to children with asthma treated with inhaled glucocorticoids affect short-term growth or bone turnover?[J]. Pediatric Pulmonology, 2003, 36(5): 399-404.

[16] Urashima M, Segawa T, Okazaki M, et al. Randomized trial of vitamin D supplementation to prevent seasonal influenza A in schoolchildren[J]. The American Journal of Clinical Nutrition, 2010, 91(5): 1255-1260.

[17] Majak P, Olszowiec-Chlebna M, Smejda K, et al. Vitamin D supplementation in children may prevent asthma exacerbation triggered by acute respiratory infection[J]. The Journal of Allergy and Clinical Immunology, 2011, 127(5): 1294-1296.

[18] Lewis E, Fernandez C, Nella A, et al. Relationship of 25-hydroxyvitamin D and asthma control in children[J]. Annals of Allergy, Asthma & Immunology: Official Publication of the American College of Allergy, Asthma, & Immunology, 2012, 108(4): 281-282.

[19] Yadav M, Mittal K. Effect of vitamin D supplementation on moderate to severe bronchial asthma[J]. Indian Journal of Pediatrics, 2014, 81(7): 650-654.

[20] Darabi B, Moin M, Purpbak Z. The effect of vitamin D supplementation over asthma outcome[J]. Iran J Allergy Asthma Immunol, 2013, 12: S87.

[21] Majak P, Rychlik B, Stelmach I. The effect of oral steroids with and without vitamin D3 on early efficacy of immunotherapy in asthmatic children[J]. Clinical and Experimental

Allergy: Journal of the British Society for Allergy and Clinical Immunology, 2009, 39(12): 1830-1841.
[22] Baris S, Kiykim A, Ozen A, et al. Vitamin D as an adjunct to subcutaneous allergen immunotherapy in asthmatic children sensitized to house dust mite[J]. Allergy, 2014, 69(2): 246-253.
[23] Jat K R, Goel N, Gupta N, et al. Efficacy of vitamin D supplementation in asthmatic children with vitamin D deficiency: A randomized controlled trial (ESDAC trial)[J]. Pediatric Allergy and Immunology: Official Publication of the European Society of Pediatric Allergy and Immunology, 2021, 32(3): 479-488.
[24] Thakur C, Kumar J, Kumar P, et al. Vitamin-D supplementation as an adjunct to standard treatment of asthma in children: A randomized controlled trial (ViDASTA Trial)[J]. Pediatric Pulmonology, 2021, 56(6): 1427-1433.
[25] Kerley C P, Hutchinson K, Cormican L, et al. Vitamin D3 for uncontrolled childhood asthma: A pilot study[J]. Pediatric Allergy and Immunology: Official Publication of the European Society of Pediatric Allergy and Immunology, 2016, 27(4): 404-412.
[26] Ducharme F M, Jensen M, Mailhot G, et al. Impact of two oral doses of 100,000 IU of vitamin D3 in preschoolers with viral-induced asthma: a pilot randomised controlled trial[J]. Trials, 2019, 20(1): 138.
[27] Forno E, Bacharier L B, Phipatanakul W, et al. Effect of Vitamin D3 Supplementation on Severe Asthma Exacerbations in Children With Asthma and Low Vitamin D Levels: The VDKA Randomized Clinical Trial[J]. JAMA, 2020, 324(8): 752-760.
[28] Aglipay M, Birken C S, Parkin P C, et al. Effect of High-Dose vs Standard-Dose Wintertime Vitamin D Supplementation on Viral Upper Respiratory Tract Infections in Young Healthy Children[J]. JAMA, 2017, 318(3): 245-254.
[29] Jensen M E, Mailhot G, Alos N, et al. Vitamin D intervention in preschoolers with viral-induced asthma (DIVA): a pilot randomised controlled trial[J]. Trials, 2016, 17(1): 353.
[30] Tachimoto H, Mezawa H, Segawa T, et al. Improved control of childhood asthma with low-dose, short-term vitamin D supplementation: a randomized, double-blind, placebo-controlled trial[J]. Allergy, 2016, 71(7): 1001-1009.
[31] Najmuddin F, Lahiri K. Vitamin D in Pediatric Asthma and Allergic Rhinitis: Benefits beyond Skeletal Health[J]. Insights in Allergy, Asthma & Bronchitis, 2017, 3(1).
[32] Bar Yoseph R, Livnat G, Schnapp Z, et al. The effect of vitamin D on airway reactivity and inflammation in asthmatic children: A double-blind placebo-controlled trial[J]. Pediatric Pulmonology, 2015, 50(8): 747-753.
[33] Swangtrakul N, Manuyakorn W, Mahachoklertwattana P, et al. Effect of vitamin D on lung function assessed by forced oscillation technique in asthmatic children with vitamin D deficiency: A randomized double-blind placebo-controlled trial[J]. Asian Pacific Journal of Allergy and Immunology, 2022, 40(1): 22-30.
[34] Kang Q, Zhang X, Liu S, et al. Correlation between the vitamin D levels and asthma attacks in children: Evaluation of the effects of combination therapy of atomization inhalation of budesonide, albuterol and vitamin D supplementation on asthmatic patients[J]. Experimental and Therapeutic Medicine, 2018, 15(1): 727-732.
[35] Rosser F J, Han Y Y, Forno E, et al. Effect of vitamin D supplementation on total and allergen-specific IgE in children with asthma and low vitamin D levels[J]. The Journal of Allergy and Clinical Immunology, 2022, 149(1): 440-444.e2.
[36] Aglipay M, Birken C, Dai D, et al. 70 high dose vitamin D for the prevention of wheezing in preschoolers: a secondary analysis of a randomized clinical trial[J]. Paediatrics and Child Health, 2019, 24 (S2): e27-8.
[37] Jerzynska J, Stelmach W, Rychlik B, et al. The clinical effect of vitamin D supplementation combined with grass-specific sublingual immunotherapy in children with allergic rhinitis[J]. Allergy and Asthma Proceedings, 2016, 37(2): 105-114.
[38] Chen J, Ren Y, He N, et al. A randomized controlled study of vitamin D3 supplementation on childhood asthma control[J]. Chongqing Medicine, 2017, 46 (32): 4505-4507, 4510.
[39] A. El-Korashi L, E. Nafea O, G. Zake L, et al. Effect of Vitamin D Adjuvant and Allergen

Specific Immunotherapy on Serum IL-10 and IL-17 Levels in Childhood Asthma: A Controlled Clinical Trial[J]. Egyptian Journal of Medical Microbiology, 2021, 30(1): 175-181.
[40] Litonjua A A, Carey V J, Laranjo N, et al. Six-Year Follow-up of a Trial of Antenatal Vitamin D for Asthma Reduction[J]. The New England Journal of Medicine, 2020, 382(6): 525-533.
